# Early Surgery Is Feasible for a Very Large Congenital Infantile Fibrosarcoma Associated With Life Threatening Coagulopathy: A Case Report and Literature Review

**DOI:** 10.3389/fped.2019.00529

**Published:** 2019-12-19

**Authors:** Hamidah Alias, Abdul Halim Abdul Rashid, Sie Chong Doris Lau, C-Khai Loh, Jamari Sapuan, Sharaf Ibrahim, Reena R. Md Zin, Yock Ping Chow, Hirokazu Kanegane, Mariko Eguchi

**Affiliations:** ^1^Department of Pediatrics, The National University of Malaysia, Kuala Lumpur, Malaysia; ^2^UKM Medical Molecular Biology Institute, The National University of Malaysia, Kuala Lumpur, Malaysia; ^3^Department of Orthopedic and Traumatology, The National University of Malaysia, Kuala Lumpur, Malaysia; ^4^Department of Pathology, Faculty of Medicine, The National University of Malaysia, Kuala Lumpur, Malaysia; ^5^Department of Pediatrics and Developmental Biology, Graduate School of Medical and Dental Sciences, Tokyo Medical and Dental University, Tokyo, Japan; ^6^Department of Pediatrics, Graduate School of Medicine, Ehime University, Toon, Japan

**Keywords:** congenital infantile fibrosarcoma, coagulopathy, infantile tumor, surgery, chemotherapy

## Abstract

**Background:** Congenital infantile fibrosarcoma (CIF) is a rare malignant soft tissue tumor that predominantly occurs in children under 1 year of age. CIF is frequently misdiagnosed with other conditions like hemangioma of infancy, infantile fibromatosis, or kaposiform hemangioendothelioma. Disseminated intravascular coagulopathy (DIVC) is rarely reported to be associated with CIF.

**Case presentation:** We describe an infant who presented with a large mass over the right arm. She was initially treated conservatively as hemangioma but was later confirmed by tissue histopathological examination to have CIF as the mass rapidly increased in size. She developed massive intra-tumoral bleed with DIVC whilst receiving neoadjuvant chemotherapy requiring multiple blood products transfusion. An urgent near-total resection of the tumor was performed in view of life threatening bleeding despite multiple blood transfusions. Post-operatively, she received further adjuvant chemotherapy. Subsequently, she remained in complete remission 32 months off-treatment and has full function of the affected limb.

**Conclusions:** CIF is an important condition to be considered in infant who has large mass over the extremity. DIVC could be associated with large CIF and when it occurs can be life-threatening. Whenever feasible early surgery should be performed in very young patients with large CIF to prevent mortality from bleeding.

## Background

A large upper extremity mass in an infant can be very challenging to diagnose as several conditions could have similar clinical appearance. Differential diagnoses include hemangioma of infancy, lymphatic malformation, rapidly involuting congenital hemangioma, kaposiform hemangioendothelioma, congenital fibrosarcoma, infantile myofibromatosis, primary myxoid mesenchymal tumor of infancy, and some other rare tumors (1–5). Congenital infantile fibrosarcoma (CIF) is a rare pediatric soft-tissue sarcoma and is typically observed in children <1 year of age (6–8). Although it is locally aggressive, metastasis is rare. A recent finding of translocation *t*(12;15)(p13;q25) between the ETV6 gene and the NTRK3 gene has been specifically associated with CIF (9, 10). ETV6-NTRK3 fusion occurs early in the oncogenesis of CIF, thus provides a useful marker for diagnosis. Massive bleeding or disseminated intravascular coagulopathy (DIVC) associated with CIF could be a feature in the very young infant, and requires urgent intervention (11). We report an infant with a large CIF of the right arm who developed DIVC during preoperative chemotherapy. The infant underwent urgent surgery to prevent mortality from the massive bleeding.

## Case Presentation

**Figure 1:**
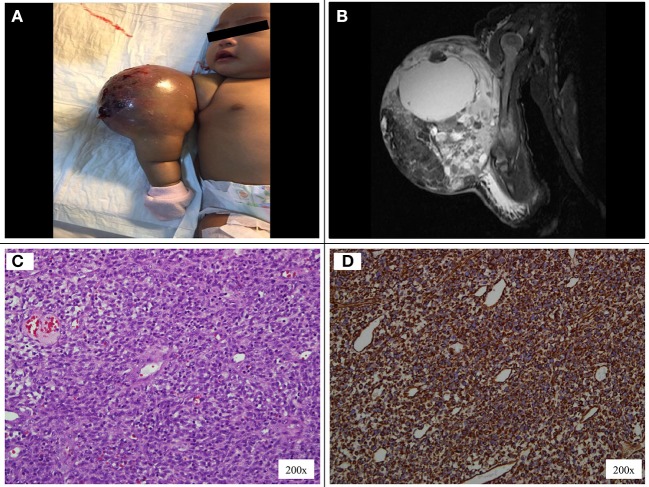
**(A)** Vascular-appearing mass with bluish discoloration of the overlying skin at the right arm. **(B)** MRI T1-weighted image shows a large, well-defined, lobulated intramuscular mass at the mid and distal right arm, measuring 5.7 × 5.3 × 6.8 cm. **(C)** The tumor cells display uniform, round, oval to spindle shaped hyperchromatic nuclei and scanty cytoplasm. **(D)** On immunohistochemical staining the tumor cells are positive to vimentin.

A 7-week-old full-term girl presented with a large mass over the right arm. The mass was first noticed at birth to be a 2 × 2-cm in size and was treated conservatively as congenital hemangioma. However, at 5 weeks old, the mass had rapidly increased in size. On physical examination, the patient had a firm, non-pulsatile, vascular-appearing mass, 6 × 5 × 7-cm in size, with an overlying skin of bluish discoloration and a few bleeding spots (Figure 1A). There was no lymph node swelling in the right upper arm, cubital fossa, or axilla. MRI revealed a large, well-defined intramuscular mass at the mid and distal right arm, predominantly iso-intense to muscle on T1-weighted sequence, and heterogeneously hyperintense on T2-weighted sequence (Figure 1B). The MRI findings were interpreted as being consistent with a bleeding intramuscular infantile hemangioma, and she was observed without any treatment. However, the mass continued to grow rapidly, hence a biopsy was performed to ascertain the diagnosis. The histopathological examination showed dense cellular neoplastic spindle cells arranged in short interlacing fascicles with mild pleomorphism, and frequent mitoses (Figure 1C). Some areas with prominent hemangiopericytoma-like vascular pattern were observed. Immunohistochemical stains were negative for desmin, muscle-specific actin, myoD1, CD34, BCL-2, S100, and positive for vimentin (Figure 1D). Based on these findings, a diagnosis of CIF was made. A complete staging which included CT scan of the chest and abdomen, bone scan, and bilateral bone marrow aspirate and trephine was performed and revealed no evidence of metastasis.

**Figure 2:**
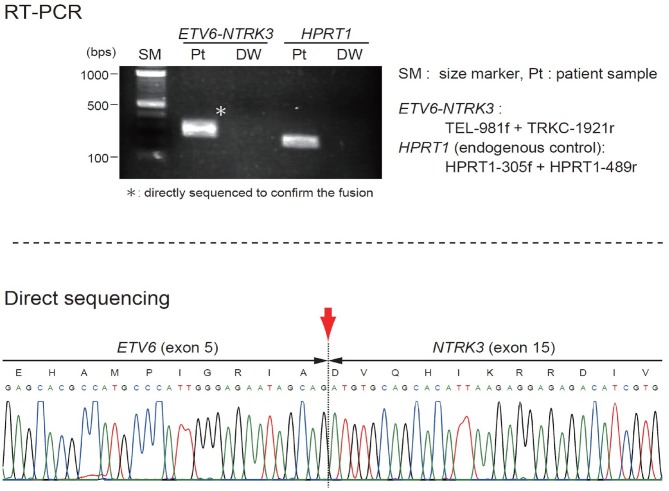
Sequence analysis for ETV6-NTRK3 fusion.

The patient was started on VAC chemotherapy, modified at 50% of the usual dose (vincristine: 0.025 mg/kg/dose, actinomycin D: 0.025 mg/kg/dose) without an alkylating agent. Pre-chemotherapy assessment revealed normal cardiac, renal and liver function. Her initial full blood count also showed normal hemoglobin and platelet count. During the 2nd week of chemotherapy, she developed massive intra-tumoral bleed with DIVC; the mass size had increased markedly. A repeat MRI showed increased mass size to 10.3 × 11.3 × 12.3-cm with intra-tumoral hemorrhage. The coagulation parameters showed prothrombin time of 18.3 s with INR ratio of 1.57, activated prothrombin time (APTT) of 61.2 s with APTT ratio of 1.58, fibrinogen level of 0.6 g/l, and D-dimer of 19.21 ug/ml. She required multiple platelet, fresh frozen plasma (FFP), cryoprecipitate and red blood cells transfusions. Despite multiple blood products transfusion the DIVC parameters did not improve significantly and the hemoglobin level dropped to lowest of 5.7 g/dL and platelet of 21 × 10^9^/L. Clinically, continuous blood oozing from few bleeding spots on the tumor surface was observed. At this juncture a multidisciplinary discussion was made to decide on life-saving tumor resection. A high risk informed consent explaining on the risk of right upper limb amputation and mortality from profuse bleeding intraoperatively was obtained from the parents. An urgent angiogram was then performed and embolization of 2 branches of the posterior circumflex right humeral artery was performed before proceeding to tumor excision. Intra-operatively, the tumor measured 16 × 16-cm and involved the lateral head of triceps and lateral side of biceps. There was bleeding hematoma within the tumor. She required multiple blood products transfusion throughout the surgery; 50 mls/kg of packed red blood cells transfusion, 30 mls/kg of FFP transfusion, 15 mls/kg of platelet and cryoprecipitate transfusions, respectively. The orthopedic surgeons were able to salvage the limb by performing near-total resection of the tumor. The radial nerve which was entrapped within the tumor was preserved. Post-operatively the patient's hemodynamic status was stable and the hemoglobin level had increased to 7.9 mg/dL and platelet of 243 × 10^9^/L. The DIVC resolved within 24 h post-operatively and there was no inotropic support needed. An ETV6(TEL)-NTRK3(TRKC) transcript was detected by reverse transcriptase-polymerase chain reaction, indicating the molecular diagnosis of CIF (Figure 2). Subsequent MRI showed residual tumor in the biceps brachii (anterior lateral) and in the long head of triceps. She completed another 5 cycles of chemotherapy post-operatively. MRI performed at the end of treatment, 24 and 30 months off treatment revealed no residual lesion. Currently she is almost 3 years off treatment and has remained well. She has full function of the right limb.

## Discussion

CIF is a rapidly growing tumor, and in a few weeks or months could become highly vascular. The extremities are affected in most cases of CIF, followed by the head, neck, and trunk. Despite being locally aggressive, CIF is associated with a high long-term survival rate. Surgical excision is the primary treatment modality and, neoadjuvant chemotherapy should be considered in unresectable cases or to reduce morbidity from surgery given the chemosensitive nature of the tumor (12). Bleeding or consumptive coagulopathy is a rare event in CIF and can be life-threatening. Most literature reported the occurrence of massive bleeding or coagulopathy in patients diagnosed within the first 2 months of life, which led to mortality in some patients (11, 13). Many of the patients were initially treated as hemangioma of infancy. In our patient, neoadjuvant chemotherapy was given with the aim to prevent limb amputation or mutilating surgery; however, she developed life threatening DIVC while on initial treatment. DIVC complication was previously observed mainly in very young patients with CIF (Table 1). The pathophysiology of consumptive coagulopathy in CIF is not well-understood. However, a similar phenomenon to Kasabach-Merritt syndrome (hemolytic anemia, thrombocytopenia, and secondary consumptive coagulopathy) which occurred in other vascular tumors could be the basis given the high vascularity of CIF. In our case, early surgery of the huge tumor had successfully prevented life threatening complication from DIVC while the patient received neoadjuvant chemotherapy.

**Table 1:** Case report of CIF associated with DIVC.

**References**	**Gender**	**Age**	**Location**	**Size, cm**	**Surgical procedure**	**Chemotherapy**	**Outcome**
Salman et al. (11)	Male	2 months	Dorsum right hand	5.2 × 4.7 × 1.3	Subtotal resection	Yes, VAC; 8 cycles	In remission at 3 years off treatment
	Male	2 months	Left arm	8.3 × 5 × 2.7	Gross resection	Neo-adjuvant, VAC; 2 cycles	In remission at 3 years off treatment
Duan et al. (8)	Male	4 days	Left forearm, recurrent	8 × 7 × 6	Resection; Amputation at supracondylar level at recurrence	No	In remission
Kraneburg et al. (13)	Male	Newborn	Left leg	11.8 × 9.3 × 8.5	Resection; through-the-knee amputation	No	In remission at 2 years old
Kerl et al. (2)	Female	Newborn	Left elbow	10 (in diameter)	Resection	Yes, VAC; 9 cycles	In remission at 4 years old
Dumont et al. (14)	Male	Prenatal	Right leg	10.7 × 7.3 × 8.6	Leg amputation	No	Died at day 8 of life
Muzaffar et al. (15)	Female	Prenatal	Left hand	5.4 × 7.8 × 4.0	Resection	Yes, neo-adjuvant, VAC; 2 cycles	In remission at 22 months old
Asgari et al. (4)	Female	Newborn	Left palm	Grapefruit-sized	Resection	Yes, neo-adjuvant, VAC	In remission at 16 months old
Boon et al. (16)	Female	Newborn	Right cervico-occipital region	Same size of her cranium	Resection	Yes, VCR, adriamycin, cyclophosphamide	In remission at 2 years old
	Male	Newborn	Right scapula	9 × 8.5 × 4.5	Resection	N/A	In remission at 1 year old
Walton et al. (17)	Male	Prenatal	Chest wall	8 × 8	Unresectable	No	Died at 24 h of life
Edwards et al. (18)	Male	Newborn (premature)	Left sacrococcygeal to left extremity	14.5 × 11.0	Debulking	Yes, VCR, dactinomycin	Died at day 22 of life

*VAC, vincristine, dactinomycin, cyclophosphamide; VCR, vincristine*.

## Conclusion

Bleeding or DIVC is a rare manifestation of CIF and it could be associated with a large mass in the extremities, trunk, intrathoracic, or intraabdominal. Whenever feasible early surgery should be performed in very young patients with large CIF at risk of life threatening bleeding to prevent mortality.

## Data Availability Statement

All datasets generated for this study are included in the article/supplementary material.

## Ethics Statement

The National University of Malaysia (UKM) Research Ethics Committee has approved the study. The Research Ethics Committee, The National University of Malaysia operates in accordance to the International Conference of Harmonization Good Clinical Practice Guidelines. Parental written consent has been obtained allowing inclusion of material pertaining to the patient. The patient's parents were informed that no identifying information will be published and this has been acknowledged by them.

## Author Contributions

HA acquired the clinical data and drafted the manuscript. HA, AA, SL, C-KL, JS, and SI were responsible for the clinical management of the patients. RZ and YC were responsible for the pathological diagnosis. ME was responsible for the molecular test and diagnosis. HK and ME were responsible for interpretation of the molecular diagnosis and critical revision of the manuscript. All authors have read and approved the final manuscript.

### Conflict of Interest

The authors declare that the research was conducted in the absence of any commercial or financial relationships that could be construed as a potential conflict of interest.

## Abbreviations

CIF: Congenital infantile fibrosarcoma
DIVC: Disseminated intravascular coagulopathy
MRI: Magnetic resonance imaging
CT: Computerized tomography
MAG3: Mercaptoacetyltriglycine.
